# Supplementary material for: Learning curve and surgical outcome of robotic assisted colorectal surgery with ERAS program
Source: Sci Rep. 2022 Nov 29;12:20566. doi: 10.1038/s41598-022-24665-w (PMC9709162; doi:10.1038/s41598-022-24665-w)
Supplement: Supplementary file 1 — Supplementary Information 1. [file 41598_2022_24665_MOESM1_ESM.zip › LSC Raw data-Ñ[▒K/083/N_ñw╝╨░O▒Kñσ.pdf]

W92 / W92

臺中榮民總醫院
手術室護理記錄

[redacted]

基本資料

- 室別： 二醫手術室-32室，手術科別： CRS，手術類別： 常規，日期： 2020/07/09
- 進入時間： 1450 ； 麻醉開始時間： 1450 ； Time Out時間： 1545 ； 劃刀時間： 1546 ； 手術結束時間： 1830 送出時間： 1900 ；
- 麻醉方式： GE

  診斷：RECTAL PROLPASED

  手術名稱：Laparoscopic Anterior resection and anastomosis-benign

  手術局部用藥：nil

  術前預防性抗生素：
  - 抗生素名稱一： Veterin 1GM IVD/由麻醉護理師給予
  - 時間： 1530
  - 上次簽章: 蔡易軒

  ，追加預防性抗生素：
- 手術醫師： 蔣鋒帆，林昌霖
- 麻醉醫師： VS 賴慧卿
-
  - 刷手護士A ： 詹佳柔，時間： 1450-1730，接替刷手一： 王毓均，時間： 1730-1830，接替刷手二： 林宜旻，時間： 1830-1900
  - 流動護士A ： 蔡易軒，時間： 1450-1800，接替流動一： 詹佳柔，時間： 1800-1900

皮膚狀況

A-V Shunt： 無

術前： 完整

記錄者： 上次簽章: 蔡易軒

術後： 完整

記錄者： 詹佳柔

手術準備

手術臥式 單一手術臥式

-
  - 手術臥式： 截石臥式
  - 使用附件： 腳架
  - 皮膚準備： Hibiscrub，其它： 2%CHLORHEXIDINE
  - 電燒： 使用電燒: 電極片位置： 左背部，其它： 電燒墊，右背部，其它： 電燒墊，記錄者： 上次簽章: 蔡易軒

止血帶T： 無

植入物

- 植入物名稱/號碼/數量/部位： ｓｕｒｇｉｗｒａｐ　１００ｘ１３０ｍｍ／壹／ｐｅｌｖｉｃ

，記錄者： 詹佳柔

標本送檢

- 細菌培養 無
- 細胞學 無
- 冰凍切片 無
- 病理標本

  組織名稱一：S COLON
- 截肢 無

標本交付 標本： 1 袋，標本： 0 盒，標本： 0 瓶，細胞學： 0 管，Culture Tube： 0 管，被交付醫師： CR 林昌霖，

- 記錄者： 詹佳柔
- 時間： 1900

傷口術後敷料

傷口敷料： 傷口敷料： 無

- 請於圖上標示部位：

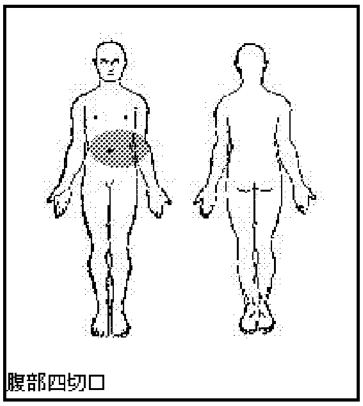

- 傷口敷料： MEPORE

導管

導管	天數	導管資訊	插入日期	插入時間	更換日期	拔除日期	拔除時間	拔除原因	預計拔除/更換	預計拔除/更換日期
Penrose Drain(1)	1	號碼:12 mm.; 部位:骨盆腔; 註記:左腹;	2020/07/09	1700						
Foley(1)	1	號碼:16 Fr.; 部位:膀胱; 註記:balloon 10cc;	2020/07/09	1520						

用物計數

- 手術中出血量： MINIMAL c.c.
- 手術中尿量： 200 c.c.
-
    - 其它： 無

用物計數： 多切口同時縫合

- 1.器械： 正確，2.縫針： 正確，3.刀片： 正確，4.紗布： 正確，5.腹部墊： 未使用，6.紗球： 未使用，7.PEANUTS ： 未使用，8.紗條： 未使用，9.絲帶： 未使用，10.針頭： 未使用，11.Vessel loop： 未使用，12.Boots： 未使用，13.Jelco： 未使用，14.棉條： 未使用，
    - 第一次：
        - 刷手護士 ： 王毓均
        - 流動護士： 詹佳柔
        - 時間： 1730

  ,
    - 第二次：
        - 刷手護士 ： 王毓均
        - 流動護士： 詹佳柔
        - 時間： 1805

  ,
    - 第三次：
        - 刷手護士 ： 王毓均
        - 流動護士： 詹佳柔
        - 時間： 1820

備註

備註： NIL

- 記錄者： 詹佳柔
- 時間： 1900

填寫護理人員 詹佳柔

正式摘要

W92 / W92

doctorRank:B
